# Red Bull Energy Drink Impact on Salivary Glands in Wistar Rats: Can Blueberry Extract Reverse the Damage?

**DOI:** 10.3390/nu16172958

**Published:** 2024-09-03

**Authors:** Samar A. Alghamdi, Emad A. Hindi, Layla Abuljadayel, Hanadi Alwafi, Amina M. Bagher, Sahar Khunkar, Nadia Bakhsh, Soad Ali, Linda Mirza, Aziza R. Alrafiah, Nimah I. Alsomali

**Affiliations:** 1 Department of Oral Biology, Faculty of Dentistry, King Abdulaziz University, Jeddah 21589, Saudi Arabia; samalgamdi@kau.edu.sa; 2 Department of Clinical Anatomy, Faculty of Medicine, King Abdulaziz University, Jeddah 21589, Saudi Arabia; eahindi@kau.edu.sa; 3 Neuroscience and Geroscience Research Unit, King Fahd Medical Research Center, King Abdulaziz University, Jeddah 21589, Saudi Arabia; 4 Department of Dental Public Health, Faculty of Dentistry, King Abdulaziz University, Jeddah 21589, Saudi Arabia; labuljadayel@kau.edu.sa; 5 Department of Pediatric and Prevention Dentistry, Batterjee Medical College, Jeddah 21442, Saudi Arabia; hanadi.alwafi@bmc.edu.sa; 6 Department of Pharmacology and Toxicology, Faculty of Pharmacy, King Abdulaziz University, Jeddah 21589, Saudi Arabia; abagher@kau.edu.sa; 7 Department of Restorative, King Abdulaziz University Hospital, Jeddah 21589, Saudi Arabia; skhenkar@kau.edu.sa; 8 AGD Department, King Abdulaziz University Hospital, Jeddah 21589, Saudi Arabia; nibakhs@kau.edu.sa; 9 Department of Histology and Cell Biology, Faculty of Medicine, Assuit University, Assuit 98467, Egypt; soadshaker@gmail.com; 10 King Abdullah Medical Complex, Ministry of Health, Jeddah 23816, Saudi Arabia; drlindamirza2008@gmail.com; 11 Department of Medical Laboratory Sciences, Faculty of Applied Medical Sciences, King Abdulaziz University, Jeddah 21589, Saudi Arabia; aalrafiah@kau.edu.sa; 12 Research Center, King Fahad Medical City, Riyadh 11525, Saudi Arabia

**Keywords:** energy drinks, Red Bull, blueberry, Wistar albino rats, oxidative stress, inflammation, submandibular salivary gland, histology, immunohistochemistry, α-smooth muscle actin

## Abstract

Energy drink (ED) consumption has become increasingly popular. Due to a lack of evidence, it was crucial to assess the effects of Red Bull (RB) consumption on the rat submandibular salivary gland and the potential therapeutic impact of blueberry (BB). Thirty rats were randomly assigned to five groups. Group 1 (Control) received distilled water. Group 2 (RB) received RB (10 mL/100 g/day) for 8 weeks. Group 3 (BB) rats were administered BB (500 mg/day for 8 weeks). Group 4 (RB + BB (L)) received RB for 8 weeks, and from the 5th week, were concurrently given BB (250 mg/day) for 4 weeks. Group 5 (RB + BB (H)) received RB for 8 weeks, and from the 5th week, were concurrently given BB (500 mg/day) for 4 weeks. At the end of the experiment, blood samples were collected, the animals were euthanized, and their submandibular salivary glands were harvested. Oxidative stress markers (MDA, GPx, CAT, and SOD) were assessed in both serum and tissue. Inflammatory markers (TNF-α, IL-6, and IL-10) were quantified in tissue. Submandibular gland specimens were prepared for light microscopy, and immunohistochemical staining was performed using anti-α-SMA. RB consumption resulted in a significant increase in MDA, TNF-α, IL-6, and IL-10, while GPx, CAT, and SOD levels decreased significantly. Degenerative changes in the gland’s structure were observed in the RB group. A significant increase in α-SMA immunoreaction was detected in myoepithelial cells. Administration of BB, particularly at a high dose, ameliorated the aforementioned findings. In conclusion, blueberry administration exhibited therapeutic effects due to its antioxidative and anti-inflammatory properties.

## 1. Introduction

Energy drinks (EDs) have become increasingly popular beverages consumed by individuals seeking a rapid energy boost and improved alertness. Studies have reported high prevalence rates of energy drink consumption among adolescents and young adults, with various factors influencing their consumption patterns. In the United States, for example, the National Center for Complementary and Integrative Health reported that about 25 percent of college students consumed energy drinks in 2018 [1]. Locally, in 2022 in Saudi Arabia, there was a high prevalence, around 74.88%, of energy drink consumption among medical students at Taif University. Red Bull (RB) is one of the most popular energy drink brands in Saudi Arabia [2].

Energy drinks typically contain high levels of caffeine, sugar, artificial sweeteners, and other substances such as taurine and herbal extracts [3]. These components are believed to provide an immediate surge of energy; however, their long-term effects on health are still under investigation. Several studies have already demonstrated the potential adverse effects of these highly caffeinated beverages on overall health, including cardiovascular issues [4], increased blood pressure [5], and insufficient sleep hours among adolescents [6].

Long-term Red Bull energy drink use has been linked to a number of systemic consequences that affect different organ systems. Long-term Red Bull use can cause major cardiovascular changes, such as elevated heart rate, systolic and diastolic blood pressure, cardiac output, and biochemical and ultrastructural changes in the heart muscles [7]. These modifications may increase the risk of cardiovascular events [8]. Long-term Red Bull usage has been linked to structural damage to the liver and kidneys, renal vascular congestion, tubular damage, and hepatic abnormalities, including changes in enzyme function [9]. Long-term Red Bull drinking has been associated with changes in dopamine transmission in the brain, especially in regions linked to addiction and reward. This pattern resembles that seen with addictive drugs, suggesting a potential for dependence on the beverage [10]. Research on animals has demonstrated that extended Red Bull drinking results in notable histological damage to the testes, encompassing atrophy, and the loss of germinal epithelial cells [11].

Additionally, oral health has come under close scrutiny in research to study its association with energy drink consumption. Energy drinks’ components could pose serious risks to oral health. For instance, the low pH of some energy drinks has been shown to cause significant surface roughness and changes to tooth enamel, leading to “erosion”. The degree of damage was greater with some energy drinks [12]. High sugar content in these drinks significantly increases the risk of caries [13]. Adolescents who consume high sugar levels in sports drinks have more severe periodontal diseases [14]. Other oral health problems associated with energy drinks are still under investigation.

Due to the increased consumption of unhealthy food in modern life, some individuals choose to consume natural supplements to mitigate the negative effects associated with certain harmful substances. For example, turmeric is consumed for its anti-inflammatory and antioxidant effects [15], garlic contains sulfur compounds that support the body’s natural detoxification processes [16], and some fruits, such as blueberries (BB), contain strong antioxidants, particularly phenolic compounds, which have been associated with various health benefits, including the prevention of diseases ranging from atherosclerosis to ischemic stroke [17]. The bioactive compounds in blueberries, particularly anthocyanins, flavonoids such as quercetin, myricetin, and kaempferol, phenolic acids such as chlorogenic acid and caffeic acid, ellagic acid, resveratrol, tannins, vitamin C, and dietary fibers contribute significantly to their health benefits [18]. Therefore, adding blueberries to the diet may have tissue-protective effects in many conditions.

It is crucial to investigate further the effects of energy drinks on both general and oral health, as well as the protective action of blueberries. The goal of the current experimental research was to shed light on the functional and histological changes in the salivary gland tissues of adult male Wistar albino rats produced by Red Bull energy drink consumption and to assess the potential therapeutic effect of oral blueberry ethanolic extract administration against the harmful effects caused by Red Bull.

## 2. Materials and Methods

### 2.1. Chemicals

Blueberry powder (Vaccinium sect. Cyanococcus) was obtained from Xi’an Pin-credit Bio-tech Co. (Xi’an, China). The glutathione peroxidase (GPx) activity assay kit and rat tumor necrosis factor (TNF), interleukin-10 (IL-10), and 6 (IL-6) ELISA kits were purchased from Elabscience (Houston, TX, USA). Rat catalase (CAT), superoxide dismutase (SOD), and malondialdehyde (MDA) assay kits were acquired from Biodiagnostic (Giza, Egypt). Anti-alpha-smooth actin (α-SMA) primary antibody was obtained from Abcam (Cambridge, UK).

### 2.2. Animals

The animal experimentation in this study was conducted in accordance with an approved protocol from the Research Ethical Committee at the Faculty of Pharmacy, King Abdulaziz University (KAU), Saudi Arabia (Approval No. PH-1443-20). Adult male Wistar rats, weighing 150–250 g each, were used in this study. The rats were obtained from the Animal House of the Faculty of Pharmacy, KAU. The rats were housed in a controlled environment with a temperature ranging from 20 to 24 °C and a 12-h light–dark cycle. They were provided with ad libitum access to a standard diet and water.

### 2.3. Experimental Design

Thirty male Wistar rats were randomly assigned to five groups (six rats per group) and housed individually in separate cages. Group 1 (Control) served as the control group, where rats received only distilled water. Group 2 (RB) received Red Bull orally at a dose of 10 mL/100 g/day for 8 weeks. Group 3 (BB) received blueberry ethanolic extract orally at a dose of 500 mg/day for 8 weeks. Group 4 (RB + BB (L)) received Red Bull orally for 8 weeks, and from the beginning of the 5th week, they were concurrently administered BB ethanolic extract at a low dose of 250 mg/day for 4 weeks [19]. Group 5 (RB + BB (H)) received Red Bull orally for 8 weeks, and from the beginning of the 5th week, they were concurrently administered BB ethanolic extract at a high dose of 500 mg/day for 4 weeks [13]. All treatments were administered orally via gavage.

At the end of the experiment, the rats were fasted for 12 h. Blood samples were then collected from the retro-orbital veins using plain tubes. The collected blood samples were centrifuged for 20 min at 3000× *g* to obtain serum. All groups of rats were subsequently euthanized by cervical dislocation while under deep ether anesthesia. The submandibular salivary gland was excised and opened longitudinally (right) and transversely (left). For histological studies, a portion of the gland was immediately fixed in a 10% formalin solution for 48 h. The remaining gland samples were rapidly frozen using liquid nitrogen and stored at −80 °C, along with the serum, for subsequent biochemical marker analysis.

### 2.4. Assessment of Oxidative Stress Biomarkers

To assess the oxidative status, oxidative stress markers were evaluated in both rat serum and submandibular gland tissue. The submandibular gland tissue was homogenized in ice-cooled phosphate-buffered saline (PBS) with a concentration of 50 mM and pH 7.4 at a ratio of 1:10 (*w*/*v*). The activity of GPx was measured using a commercial kit available from Elabscience (Cat. No. 12.65-386 U). The levels of CAT, SOD, and MDA were evaluated using commercial kits available from Biodiagnostic (Cat. No. MD 2517, SD 25 21, and MD 2529, respectively), following the manufacturer’s instructions.

### 2.5. Assessment of Inflammatory Markers

The levels of the inflammatory markers, TNF-α, IL-10, and IL-6, in the submandibular gland tissue homogenate were evaluated using ELISA kits from Elabscience (Cat. No. E-EL-R2856, E-EL-R0016, and E-EL-R0015, respectively). The ELISA assays were performed according to the manufacturer’s instructions to ensure accurate measurements.

### 2.6. Histological Examination

Histological analysis was performed at the histopathology laboratory of King Abdulaziz University Hospital. After fixation in 10% formalin solution, the tissue samples were embedded in paraffin wax and sectioned into 5 μm thick slices. These sections were stained with hematoxylin and eosin (HE) and examined using a light microscope to assess their histological characteristics.

### 2.7. Immunohistochemistry of Alpha-Smooth Actin (α-SMA)

Immunohistochemical analysis of alpha-smooth actin (α-SMA) was performed on paraffin-embedded sections of submandibular salivary tissue. The sections were deparaffinized, gradually rehydrated with ethanol, and subjected to heat-induced antigen retrieval by immersion in 10 mM sodium citrate buffer (pH 6.0) for 5 min. Following a rinse with PBS, the sections underwent immunostaining procedures. To minimize non-specific binding, the slides were incubated at room temperature for 1 h using a blocking solution containing 5% bovine serum albumin (BSA) in Tris-base buffered saline (TBS). Subsequently, the sections were incubated overnight at 4 °C with an anti-α-SMA primary antibody (Catalog Number ab7817). The following day, the sections were washed with TBS and incubated with the appropriate secondary antibody. The slides were examined under a light microscope, and images were captured using a CCD camera.

### 2.8. Data Analysis

Data were presented as mean ± standard deviation (SD). One-way ANOVA followed by Tukey’s post hoc test, conducted using GraphPad Prism version 9.1 (San Diego, CA, USA), was used to compare multiple means. A *p*-value threshold of <0.05 was considered statistically significant.

## 3. Results

### 3.1. Assessment of Oxidative Stress Biomarkers

To assess the impact of Red Bull energy drink on reactive oxygen species (ROS) levels in the experimental rats, we measured the levels of oxidative stress markers (MDA) and antioxidants (GPx, CAT, and SOD) in the blood serum and submandibular salivary gland tissue.

**Figure 1:**
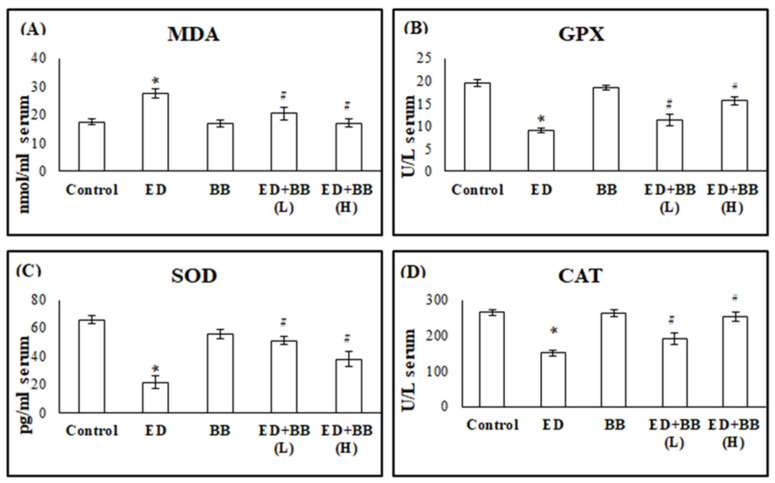
The effects of blueberry (BB) treatments on serum levels of (**A**) malonaldehyde (MDA), (**B**) glutathione peroxidase (GPx), (**C**) superoxide dismutase (SOD), and (**D**) catalase (CAT) in Red Bull (RB)-treated rats. Data are presented as mean ± SD (*n* = 6). * significant versus control *p* < 0.05, # significant versus ED *p* < 0.05.

At the serum level, administration of RB to rats in group 2 resulted in a significant increase in serum MDA levels compared to the control group (*p* < 0.05). Administration of BB alone in rats of group 3 showed no significant change in MDA levels compared to control rats. In group 4, administration of BB at a low dose level after administration of ED resulted in a significant decrease in serum MDA levels compared to the ED group (*p* < 0.05), as shown in Figure 1. Moreover, the high dose of BB administered to rats in group 5 after ED administration resulted in a significant decrease in serum MDA levels compared to the ED group (*p* < 0.01) (Figure 1).

Serum levels of GPx, CAT, and SOD were significantly decreased in rats of group 2 compared to controls (*p* < 0.05). The administration of BB alone in animals of group 3 exhibited no significant change in serum levels of GPx, CAT, and SOD compared to the control group. A low dose of BB given to rats in group 4 after the administration of ED resulted in a significant increase in serum levels of GPx, CAT, and SOD compared to group 2 (*p* < 0.05). Meanwhile, these levels were significantly increased in group 5, which received the high dose of BB, as compared to group 2 (*p* < 0.01).

**Figure 2:**
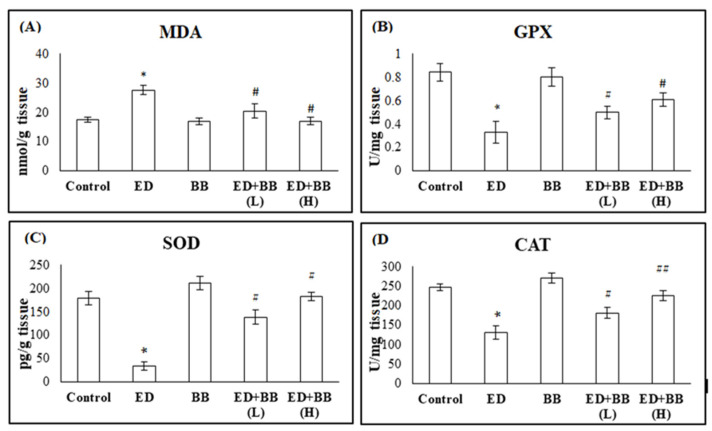
The effects of blueberry (BB) treatments on submandibular salivary gland tissue levels of (**A**) malonaldehyde (MDA), (**B**) glutathione peroxidase (GPX), (**C**) superoxide dismutase (SOD), and (**D**) catalase (CAT) in Red Bull (RB)-treated rats. Data are presented as mean ± SD (*n* = 6). * significant versus control *p* < 0.05. # significant versus ED *p* < 0.05. ## significant versus ED *p* < 0.01.

At the submandibular salivary gland tissue level, the administration of RB to rats of group 2 resulted in a significant increase in tissue MDA levels compared to the control group (*p* < 0.05). The administration of BB alone, in animals of group 3, showed no significant change in MDA levels compared to the control group. In group 3, treatment with BB at a low dose after administration of ED resulted in a significant decrease in MDA levels in submandibular salivary gland tissue compared to the ED group (*p* < 0.05), as shown in Figure 2. Moreover, the treatment of rats in group 4 with BB at a high dose level after ED administration resulted in a significant decrease in serum MDA levels compared to the ED group (*p* < 0.01) (Figure 2).

The activity of GPx, CAT, and SOD was significantly decreased in the submandibular salivary gland tissue of rats in group 2 compared to controls (*p* < 0.05). The administration of BB alone in rats of group 3 exhibited no significant alteration in the levels of GPx, CAT, and SOD compared to control rats. Low dose BB administration to animals in group 4 exhibited a significant increase in tissue levels of GPx, CAT, and SOD compared to the ED group (*p* < 0.05). Meanwhile, these levels were significantly increased in group 5 compared to rats in group 2 (*p* < 0.01).

These findings demonstrated that the consumption of energy drinks for eight weeks increased oxidative stress (MDA) and decreased antioxidant levels (GPx, SOD, and CAT) in both blood and submandibular salivary gland tissue. Following a four-week period of blueberry administration, improvements were observed (Figure 1 and Figure 2). Moreover, the therapeutic effect of blueberry was dose-dependent, with the high dose showing a greater effect.

### 3.2. Assessment of Inflammatory Markers

To evaluate the influence of Red Bull energy drink on inflammatory marker levels in the experimental rats, we measured the levels of tumor necrosis factor (TNF), interleukin-10 (IL-10), and interleukin-6 (IL-6) in the submandibular salivary gland tissue.

**Figure 3:**
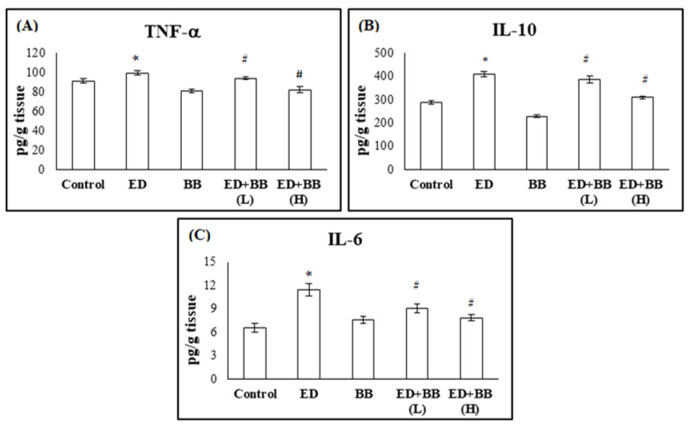
The effects of blueberry (BB) treatments on submandibular salivary gland tissue levels of (**A**) tumor necrosis factor (TNF), (**B**) interleukin-10 (IL-10), and (**C**) interleukin-6 (IL-6) in Red Bull (RB)-treated rats. Data are presented as mean ± SD (*n* = 6). * significant versus control *p* < 0.05. # significant versus ED *p* < 0.05.

Administration of RB to rats in group 2 resulted in a significant increase in TNF-α, IL-10, and IL-6 levels (*p* < 0.05) in the submandibular salivary gland tissue compared to control rats. The administration of BB alone in rats of group 3 showed no significant change in the submandibular salivary gland tissue levels of TNF-α, IL-10, and IL-6 compared to control animals. In group 4, administration of BB at a low dose after administration of ED caused a significant decrease in TNF-α, IL-10, and IL-6 levels in the submandibular salivary gland tissue compared to the ED group (*p* < 0.05), as shown in Figure 3. Moreover, the administration of a high dose of BB to rats in group 4 after ED administration resulted in a significant decrease (*p* < 0.05) in the submandibular salivary gland tissue TNF-α, IL-10, and IL-6 levels compared to the ED group (Figure 3). Meanwhile, the high dose of BB significantly increased these levels in rats of group 5 compared to group 2 (*p* < 0.01).

These findings indicated that the submandibular salivary gland tissue’s inflammatory markers increased after eight weeks of energy drink administration. However, following a four-week period of blueberry administration, improvements were observed (Figure 3). The impact of the high dose of BB was greater than that of the low dose.

### 3.3. Histological Findings

**Figure 4:**
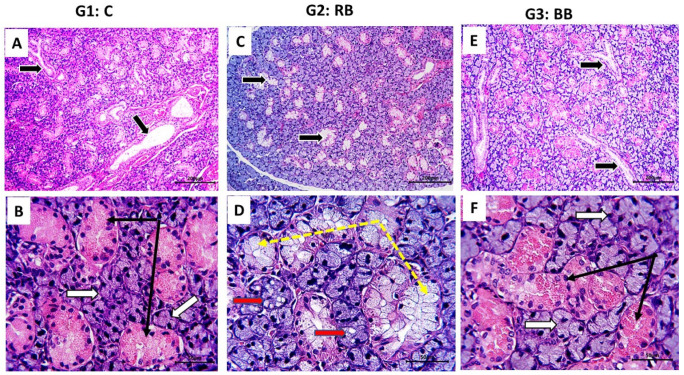
Photomicrographs of sections of rat submandibular gland of control, RB and BB groups stained with H&E to reveal its histological architecture: In control rats, normal glandular parenchyma was evident in form of acini and interlobular ducts ((**A**), arrows); granular tubules ((**B**), black arrow) with its characteristic acidophilic granules and acini ((**B**), white arrows) were clear. In RB group, some degenerative changes were recorded, granular tubules appeared as white patches with lower magnification ((**C**), arrows), due to loss of its acidophilic granules ((**D**), yellow dotted arrows); the glandular acini showed vacuolar degeneration ((**D**), red arrows). In BB group, appeared normal; interlobular ducts ((**E**), arrows), acini ((**F**), white arrows), and granular tubules with their acidophilic granules ((**F**), black arrows) were clear. Stain: H&E, Mag., (**A**,**C**,**E**) ×100; (**B**,**D**,**F**) ×400. Bar = 50 µm.

**Figure 5:**
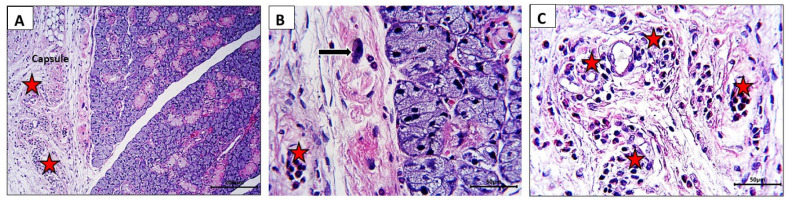
Photomicrographs of submandibular salivary gland of rats received RB revealing the histological changes in glandular capsule which showed inflammatory cell infiltration ((**A**–**C**) stars). Mast cell ((**B**), arrow) detected in capsular region. Stain: H&E. Mag. (**A**) ×100, (**B,C**) ×400. Bars = (**A**) 20 µm—(**B**,**C**) 50 µm.

In the current study, rats in both the control group and the BB group showed normal histological architecture in their submandibular glands (Figure 4A–C,E,F); normal glandular acini, ducts, and granular tubules with their characteristic acidophilic cytoplasmic granules (B,F, black arrows). On the other hand, the RB group showed marked degenerative changes manifested by loss of the acidophilic granules of the granular tubules (Figure 4D, yellow dotted arrows) and vacuolar degeneration in the acini (Figure 4D, red arrows). The glandular capsule of RB-treated rats showed inflammatory cell infiltration (Figure 5A–C, stars), in addition to the presence of solitary mast cells (Figure 5B, arrow).

**Figure 6:**
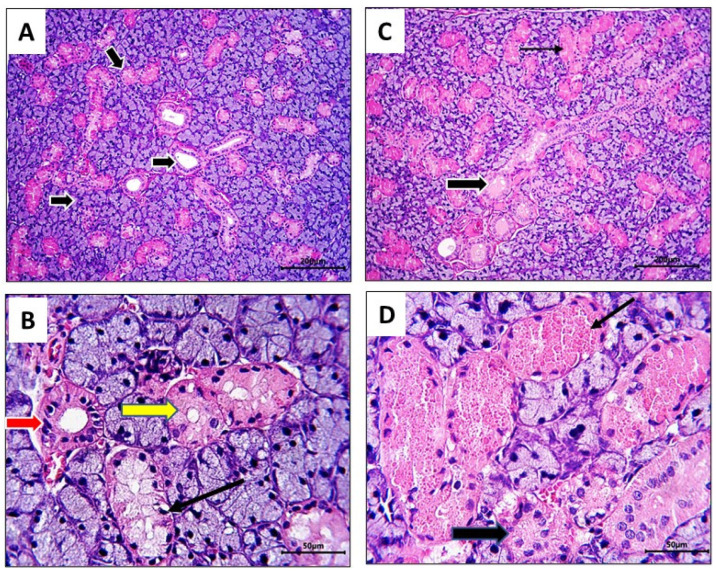
Photomicrographs of submandibular glands of rats received RB followed by BB extracts, low dose of BB given to group 4 (**A**,**B**), and high dose of BB given to group 5 (**C**,**D**). In group 4 (RB + BB (L)), glandular parenchyma was normal ((**A**), arrows; (**B**), yellow arrow), few granular tubules still lack its acidophilic granules ((**B**), black arrow) and striated duct showed pyknotic nuclei ((**B**), red arrow). In group 5 (RB +BB (H)), glandular parenchyma was normal, granular tubules ((**C**,**D**) thin arrows) preserved its acidophilic granules, and striated ducts ((**C**,**D**) thick black arrows) were normal. Stain: H&E. Mag. (**A**,**C**) ×100; (**B**,**D**) ×400. Bar = 200 µm (**A**,**C**), 50 µm (**B**,**D**).

In blueberry-treated (RB + BB) rats (group 4, low dose, and group 5, high dose), the histological profile of the glandular parenchyma was improved in group 4 (Figure 6A, arrows), although some granular tubules (Figure 6B, black arrow) still lacked their acidophilic granules and some striated ducts had pyknotic nuclei (Figure 6B, red arrows). In group 5 rats, the glandular parenchyma appeared normal (Figure 6C, arrows); the granular tubules had retained their acidophilic granules (Figure 6D, thin arrow), and striated ducts (Figure 6D, thick arrow) were also normal.

### 3.4. Immunohistochemistry of Alpha-Smooth Actin (α-SMA)

**Figure 7:**
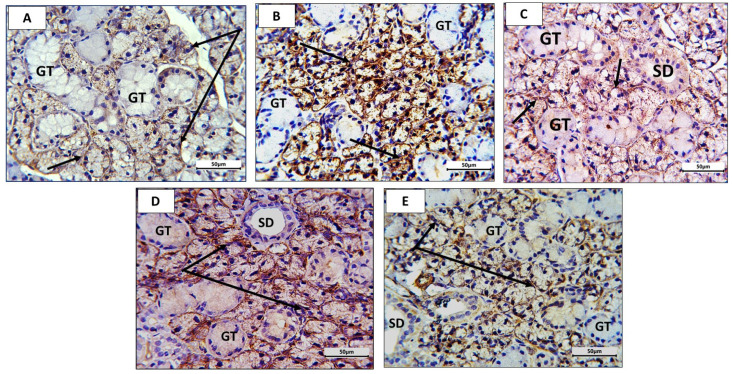
Photomicrographs of sections of rat submandibular gland of different groups imuno-stained to localize alpha-smooth muscle actin (α-SMA): In control rats (**A**) and blueberry-treated rats (**C**), α-SMA was expressed mildly in glandular acini and myoepithelial cells ((**A**,**C**) arrows) but not in granular tubules. In RB-administrated rats (group 2), α-SMA was strongly expressed in glandular acini ((**B**), arrows). In group 4 (RB +BB (L)) and G5 (RB + BB (H)) rats, α-SMA expression showed a dose-dependent decrease in immunostaining intensity compared with group 2 (RB) with milder stain in group 5 rats ((**D**,**E**) arrows). In all groups, striated ducts (SD) showed mild immunostaining in their basal part, while granular tubules (GT) were negative. Mag. ×400. Bar = 50 µm.

Alpha-smooth muscle actin (α-SMA) is normally present in the glandular parenchyma. Mild expression of α-SMA was localized in the acini and their myoepithelial cells (Figure 7A,C, arrows) in the control (group 1) and blueberry (group 3) groups. In group 2 (RB) rats, strong immunostaining was detected in the acini and myoepithelial cells (Figure 7B, arrows). In (ED + BB)-treated rats (group 4 and group 5), the intensity of α-SMA immunostaining was decreased in a dose-dependent manner, being similar to the control group in group 5 rats. In group 4 and group 5, α-SMA was localized in the acini and myoepithelial cells (Figure 7D,E, arrows). In all groups, the striated ducts (SD) showed mild immunostaining in their basal part, while the granular tubules (GT) were negative.

## 4. Discussion

The present study revealed that the consumption of Red Bull for a period of eight weeks caused elevated oxidative stress markers in both serum and submandibular salivary gland tissue. The findings indicate that Red Bull consumption resulted in a notable reduction in serum activities of GPx, CAT, and SOD, and a significant elevation in MDA levels. Meanwhile, the administration of blueberry to rats that received Red Bull led to a significant reduction in MDA levels and elevated GPx, SOD, and CAT levels.

In this respect, Al-Eryani et al. (2018) found significantly high levels of lipid peroxides in the testes of rats given energy drinks for a period of seven weeks [20]. Moreover, Abdollahi et al. (2004) stated that administration of energy drinks resulted in a significant decrease in the activity of SOD, CAT, and GPx enzymes [21]. These enzymes protect cells from oxidative damage caused by free radicals in conjunction with the non-enzymatic antioxidant system. SOD neutralizes the extremely reactive superoxide anions by converting them to hydrogen peroxide, which is then broken down into water by GPx and CAT [22]. The significant drop in serum enzyme levels, especially in rats administered energy drinks, could be the result of an increase in superoxide radicals brought on by energy drinks that surpasses the antioxidant capacity of enzymes to counteract these radicals.

Research findings indicate that elevated caffeine exposure results in a pro-oxidant state inside human cells, hence stimulating protein oxidation; conversely, minimal caffeine administration does not impact the antioxidant ability of the cells [23]. The antioxidant defense enzymatic system is weakened by caffeine, which raises free radical activity and ultimately results in oxidative stress as seen by an increase in MDA concentration [24]. Caffeine causes a significant rise in blood urea nitrogen levels, which activates xanthine oxidase and promotes the conversion of xanthine to uric acid, as well as the generation of superoxide anions and H_2_O_2_ [24]. However, other studies have demonstrated the antioxidant properties of numerous energy drink components, including taurine, ginseng, caffeine, and guarana [25].

Blueberries became introduced as a “super fruit” due to the remarkable in vitro antioxidant activity of their rich polyphenolic components [26]. Blueberries are high in antioxidants, which have anti-inflammatory characteristics. Their high antioxidant capacity could be attributed to free radical scavenging characteristics [26,27]. Blueberry intake has been shown to be beneficial in oxidative stress-related situations [26,27]. Some researchers have investigated the effect of blueberry extract on liver functions and found that blueberries protect against toxin- and d-galactosamine-induced acute damage to the liver [28]. It was previously found that blueberry consumption decreased oxidative stress and hepatic damage in hypercholesterolemic guinea pigs [29] and d-galactose-treated rats [30] by serving as an antioxidant. Furthermore, blueberries have been shown to reduce hepatocyte damage and lipid peroxidation, hence preventing and protecting against carbon tetrachloride-induced hepatic fibrosis [31]. Single phenolic compounds present in blueberries have significant antioxidant activity; however, the combined effect of phenolics may be more potent. As a result, in the current investigation, blueberry ethanolic extract was used, with doses chosen in accordance with earlier research [28,29,30].

Antioxidant polyphenols found in blueberries include anthocyanins, flavanols, and phenolic acid. Anthocyanins most likely have the largest impact on blueberry health efficiency [26]. Blueberries are rich in anthocyanins, which are powerful antioxidants capable of scavenging reactive oxygen species (ROS). This reduces oxidative stress by neutralizing free radicals, thereby preventing cellular damage. For instance, the anthocyanin malvidin-3-glucoside from blueberries has been shown to significantly reduce lipid peroxidation and ROS levels, which are critical markers of oxidative stress [32]. Blueberry components have been found to upregulate the activity of antioxidant enzymes such as superoxide dismutase (SOD) and catalase, which are crucial for detoxifying harmful superoxide radicals and hydrogen peroxide, respectively. This enzymatic boost is essential for maintaining oxidative balance and protecting tissues from oxidative damage [33]. Anthocyanin-rich diets have been found in studies to reduce the risk of myocardial infarction in females, implying that anthocyanins could serve as anti-inflammatory agents [34,35]. Anthocyanins are plant-based substances with powerful antioxidant properties that perform a variety of activities, including scavenging free radicals that exist in the body, decreasing oxidase activity, inhibiting cholesterol absorption, and lowering bad cholesterol [36,37,38]. Furthermore, blueberry anthocyanin extracts have been shown to increase SOD and CAT activity, lower MDA levels, and prevent acrylamide-induced cytotoxicity [39]. According to the preceding literature, anthocyanins may perform an antioxidant role by removing free radicals, increasing the activity of antioxidant enzymes, and lowering the damage caused by peroxidation in the body’s tissues [39].

In our study, the ameliorative and therapeutic effect of blueberry was in a concentration-dependent manner where the high dose of blueberry showed the better effect. Our findings are in accordance with those of Ogawa et al., who estimated the antioxidant activity of anthocyanin by lipid peroxidation and free radicals scavenging ability. Their results indicated that anthocyanin displayed high scavenging capacity in a concentration-dependent manner, suggesting that the protective effect of blueberry may be due to the antioxidant activity of anthocyanin [40]. It is therefore anticipated that anthocyanins will develop into natural candidate medications for the management and prevention of many chronic illnesses. Future study is still required because the molecular mechanism underlying anthocyanin antioxidant properties is yet unknown.

Our findings showed that consuming Red Bull for eight weeks increased the inflammatory markers in the submandibular salivary gland tissue. Red Bull administration in rats significantly increased tissue levels of TNF-α, IL-10, and IL-6, according to the study. Meanwhile, administering blueberry to these rats resulted in a significant drop in these levels. However, studies on health-promoting foods conducted over the last 20 years have identified numerous ways in which blueberries are bioactive and advantageous to health. Evidence from observational studies [41], clinical studies [42], animal studies [43], and in vitro studies [44] supports an improvement in anti-inflammatory biomarkers linked to blueberry consumption. Mucin-associated and other colonic microbiota may have a role in the anti-inflammatory and immunological impacts of blueberries [45], opening a new area of study for berries and health. The benefits of blueberry have been seen in both short-term [46,47,48] and long-term [49,50] human therapies, indicating a variety of possible mechanisms of action. In the current investigation, we employed two dose levels of blueberry, low and high, and it was discovered that the beneficial effect of blueberry was dose-dependent, with the high dose showing a greater benefit than the low dose. Many critical aspects remain poorly understood in health studies of blueberries. For instance, the dose dependency of clinical impacts is most likely obscure [46,51,52]. It was indicated that anthocyanin could reduce TNF-α, IL-1, and IL-6 levels, proposing that it has a possible impact on acute inflammatory illness through inflammation reduction [53].

The anti-inflammatory effects of blueberries are largely mediated by the inhibition of the NF-κB pathway, a central regulator of inflammatory responses. Blueberry anthocyanins, such as malvidin-3-glucoside, have been shown to inhibit the degradation of IκBα, thereby preventing the activation of NF-κB and subsequent transcription of pro-inflammatory genes such as TNF-α, IL-1β, and IL-6 [54,55]. Blueberries also inhibit the expression of cyclooxygenase-2 (COX-2) and inducible nitric oxide synthase (iNOS), enzymes that are upregulated during inflammation and contribute to the production of inflammatory mediators like prostaglandins and nitric oxide. This inhibition helps to reduce inflammation at the cellular level [56].

In the current study, marked degenerative changes were detected in the rat submandibular gland in G2 (Red Bull group) in the form of vacuolar degeneration in the acini, pyknotic nuclei in striated ducts, loss of the acidophilic granules of the cells of the granular ducts, and inflammatory cell infiltration; similar results were recorded by [57] in the same animals, in addition to interlobular accumulation of collagenous fibers. Although energy drinks enhance consumer activity [58], several studies pointed to their degenerative effects in a variety of organs such as a rat’s liver [59] and kidneys [60], in addition to the stomach and pancreas [61]; renal tubules were targets of energy drink adverse effects in humans [62]. The granular tubule cells of the submandibular gland are characterized by acidophilic cytoplasmic granules, which possess active growth factors [63]. We observed that the cells of granular tubules had lost their cytoplasmic granules in G2 (Red Bull group). The same finding was recorded by Amira et al. 2018 and points to glandular degeneration [57].

Excessive consumption of Red Bull as an energy drink can cause oxidative stress through its active ingredients mainly caffeine [64]. The oxidative stress induces an inflammatory response. We noticed aggregations of inflammatory cells in the glandular stroma, which was in agreement with the same finding in the rat’s submandibular gland [57]. Similar results were recorded in other organs such as kidneys [60,62]. Our results are also in agreement with other researchers who stated that sustained consumption of energy drinks causes oxidative stress and apoptosis [65].

Alpha-smooth muscle actin (α-SMA) is a normal constituent in myoepithelial cells that surround the acini. In our study, myoepithelial cells showed more intense immunostaining in the Red Bull group than the control group, which is due to the hypertrophy of these cells as oxidative stress complications [57].

As reported in [66,67], blueberries are a significant source of antioxidants. In the current study, the administration of blueberry extract treated and protected the glandular tissues against the adverse effects of Red Bull sustained consumption, with full protection obtained with a high dose of blueberry; Al-shaikh and Rajeh, 2023 obtained comparable results in rats’ testis [67]. Blueberries, often referred to as a “superfood”, have been the focus of extensive scientific research due to their rich composition of bioactive compounds, particularly anthocyanins, flavonoids, vitamins, and fiber. These compounds contribute to a wide array of health benefits, which have been substantiated by various clinical and epidemiological studies. Regular consumption of blueberries has been associated with improved cardiovascular health. Clinical studies have shown that blueberries can help reduce blood pressure, lower levels of LDL-cholesterol, and improve endothelial function. The anthocyanins in blueberries are thought to enhance nitric oxide production, leading to vasodilation and reduced arterial stiffness [68,69]. Blueberries can improve memory and slow cognitive decline in older adults and younger populations. The polyphenolic compounds in blueberries, particularly anthocyanins, have been shown to cross the blood–brain barrier and exert neuroprotective effects by reducing oxidative stress and inflammation in the brain, as well as enhancing brain plasticity and synaptic activity [70,71]. Blueberries have been found to improve insulin sensitivity, which is a critical factor in the management of type 2 diabetes. The high fiber content, along with the polyphenols in blueberries, slows down the digestion of sugars, leading to better glycemic control [44]. Blueberries may also play a role in weight management by modulating lipid metabolism and reducing inflammation in adipose tissue. This is particularly relevant in the context of diet-induced obesity, where blueberries have been shown to reduce fat accumulation and improve markers of metabolic health [18]. Blueberries have been shown to positively influence gut microbiota composition, increasing the abundance of beneficial bacteria while reducing harmful species. This modulation of the gut microbiome is associated with improved digestive health and enhanced immune function [44].

## 5. Conclusions

This study found that eight weeks of Red Bull exposure induces damage to the submandibular salivary gland tissues. Red Bull’s detrimental effects are evidently caused by increased oxidative stress, ROS generation, and inflammation, as demonstrated by the markedly elevated levels of MDA, TNF-α, IL-10, and IL-6, and the significantly decreased levels of antioxidant enzymes (GPx, CAT, and SOD) in serum and submandibular salivary gland tissue. Blueberry exhibited a significant therapeutic effect on Red Bull-induced tissue damage due to its anti-inflammatory and antioxidant capabilities, which could be attributed to the free radical scavenging properties of polyphenols, flavonoids, and other active components contained in blueberries.

Consequently, it is advised to use Red Bull with blueberries. Nevertheless, more research over prolonged periods is still required in the future, as the molecular mechanisms behind the antioxidant action of blueberries remain unclear. Furthermore, additional research is needed to bridge the gap between animal studies and human applications. This could include conducting well-designed clinical trials or observational studies in human populations, as well as exploring the use of more advanced models, such as humanized animal models or organ-on-a-chip technologies, which better mimic human physiology to explore the therapeutic effects of blueberry against Red Bull’s harmful effects. By addressing these points, this study could provide a clearer pathway for how its findings might eventually inform human health interventions.

## Data Availability

The data used to support the findings of this study are included within the article.
